# Early administration of ivabradine in patients with acute anterior ST-elevation myocardial infarction: a prospective, randomized, open-label study on in-hospital and short-term major adverse cardiac events and left ventricular function

**DOI:** 10.1186/s43044-026-00782-z

**Published:** 2026-09-22

**Authors:** Omar Mohammed Al Hawwari, Mohamed Elsayed Zahran, Khaled Mohamed Said Othman, Hazem Mansour

**Affiliations:** Ain Shams University Hospital, Cairo, Egypt

**Keywords:** Ivabradine, Anterior STEMI, Heart rate, Left ventricular ejection fraction, MACE, Cardiac remodeling, No-reflow, Arrhythmias

## Abstract

**Background:**

Elevated heart rate (HR) in acute anterior ST-elevation myocardial infarction (STEMI) increases myocardial oxygen demand and limits perfusion, worsening outcomes. Ivabradine selectively reduces HR without negative inotropy. This open-label randomized controlled study investigates the efficacy and safety of early ivabradine administration in patients with acute anterior STEMI.

**Methods:**

This randomized controlled prospective study included 300 patients with acute anterior STEMI (sinus rhythm, HR > 70 bpm) at Ain Shams University hospitals. Patients were randomized 1:1 to receive either standard beta-blocker therapy plus ivabradine (Ivabradine group, *n* = 150) or standard beta-blocker therapy alone (Control group, *n* = 150). Echocardiographic assessors and clinical event adjudicators were blinded to treatment allocation. Primary outcomes were changes in left ventricular ejection fraction (LVEF), left ventricular end-diastolic volume (LVEDV), left ventricular end-systolic volume (LVESV), HR, cardiac biomarkers (CK-MB, Troponin), and the incidence of major adverse cardiac events (MACE) at 3-month follow-up. Procedural variables (door-to-balloon time, final TIMI flow, myocardial blush grade, no-reflow phenomenon), Killip class, arrhythmic events, and guideline-directed medical therapy use were documented.

**Results:**

Baseline demographic, angiographic, and medical therapy characteristics were similar between groups. At 3 months, the Ivabradine group demonstrated a significantly greater improvement in LVEF compared to controls (mean ± SD: 51.96 ± 6.09% vs. 49.79 ± 4.47%, *p* < 0.001). LVEDV significantly decreased in the Ivabradine group (from 101.44 ± 13.84 mL to 83.65 ± 8.76 mL) but increased in the control group (from 100.49 ± 12.73 mL to 120.67 ± 10.15 mL) (*p* < 0.001 for final LVEDV between groups). Final HR was significantly lower in the Ivabradine group (62.31 ± 1.79 vs. 76.13 ± 3.54 bpm, *p* < 0.001). The incidence of heart failure hospitalization (1.3% vs. 11.3%, *p* < 0.001) and total MACE (12.7% vs. 32.0%, *p* < 0.001) were significantly reduced in the Ivabradine group. No significant differences were observed in no-reflow rates (12.0% vs. 14.7%, *p* = 0.491) or ventricular arrhythmias (2.7% vs. 4.0%, *p* = 0.523) between groups.

**Conclusion:**

Early administration of ivabradine in patients with acute anterior STEMI and elevated HR **without cardiogenic shock** was associated with better heart-rate control and favorable short-term echocardiographic outcomes, while the clinical outcome findings require confirmation in prospectively registered, adequately powered multicenter trials. **These findings apply to Killip class I-III patients; Killip class IV patients (cardiogenic shock) were excluded from this study.**

**Supplementary Information:**

The online version contains supplementary material available at https://doi.org/10.1186/s43044-026-00782-z.

## Introduction

Acute ST-elevation myocardial infarction (STEMI), particularly anterior wall STEMI, carries a significant risk of left ventricular (LV) dysfunction, remodeling, and subsequent heart failure (HF) [1]. Elevated heart rate (HR) in the acute phase is a critical pathophysiological variable that increases myocardial oxygen demand, reduces diastolic coronary perfusion time, and is an independent predictor of adverse outcomes [2].

Current guidelines mandate early revascularization and recommend oral beta-blockers within 24 h to reduce myocardial oxygen consumption and the risk of ventricular arrhythmias [3]. However, beta-blockers are limited by their negative inotropic and chronotropic effects, often leading to hypotension, fatigue, and contraindications in patients with reactive airway disease [4].

Ivabradine is a selective inhibitor of the cardiac pacemaker If current in the sinoatrial node, reducing HR without affecting myocardial contractility, blood pressure, or intracardiac conduction [5]. While its benefits are established in chronic HF with reduced ejection fraction (HFrEF) and stable coronary artery disease, its role in the acute phase of anterior STEMI is less defined [6].

This randomized controlled study aimed to investigate the efficacy and safety of early ivabradine administration in patients with acute anterior STEMI, specifically its impact on infarct size surrogates, HR control, LV function, remodeling, arrhythmic events, no-reflow phenomenon, and short-term major adverse cardiac events (MACE).

## Methods

### Study design and population

This prospective, randomized, open-label study was conducted at the Cardiology Department of Ain Shams University Hospitals, from June 2024 to February 2025. The first patient was enrolled on June 15, 2024; the final patient was enrolled on December 20, 2024; and final follow-up was completed on March 20, 2025. The investigators who performed echocardiographic assessments were blinded to treatment allocation to minimize measurement bias. Clinical outcome adjudication was performed by an independent events committee blinded to group assignment.

### Clinical trial registration

This trial was not prospectively registered. The study was initiated prior to the authors' awareness of the requirement for prospective registration of interventional studies. At the time the study commenced, institutional practice did not mandate pre-enrollment registration. The study protocol and statistical analysis plan are available upon request from the corresponding author. The journal will determine whether the lack of prospective registration affects publication eligibility.

### Inclusion criteria

- Age > 18 years
- Diagnosed with acute anterior STEMI (symptoms + ST-segment elevation in ≥ 2 contiguous anterior leads)
- Sinus rhythm with HR > 70 bpm
- Killip class I-III at presentation

### Exclusion criteria

- Bradyarrhythmia, second- or third-degree atrioventricular block
- Atrial fibrillation or flutter
- Cardiogenic shock (systolic BP < 90 mmHg, Killip class IV)
- Pregnancy or refusal to participate
- Known hypersensitivity to ivabradine
- Severe hepatic impairment

### Randomization and allocation concealment

A total of 300 patients were randomized 1:1 using a computer-generated sequence into two groups. The randomization sequence was generated by an independent statistician using a random number generator with a 1:1 allocation ratio. Permuted block randomization with block sizes of 4 and 6 was used to ensure balance. No stratification was applied. Allocation concealment was maintained using sequentially numbered, opaque, sealed envelopes prepared by the independent statistician. Patients were enrolled by the admitting cardiology resident on duty, who then opened the next sequentially numbered envelope to assign the participant to their study group. Investigators and clinical staff could not access upcoming assignments. The randomization list was securely stored and accessible only to the independent statistician.

- **Ivabradine Group (*n* = 150)**: Received standard guideline-directed therapy for STEMI plus oral ivabradine. Ivabradine was initiated in the emergency room at 2.5 mg twice daily, titrated based on resting HR to a maximum of 7.5 mg twice daily, aiming for a HR of 50–60 bpm. Ivabradine was administered within 30 min of arrival to the emergency department, prior to or during primary PCI.
- **Control Group (*n* = 150)**: Received standard guideline-directed therapy for STEMI alone.

Standard therapy included urgent reperfusion via primary percutaneous coronary intervention (PCI), dual antiplatelet therapy (aspirin + P2Y12 inhibitor), statins, ACE inhibitors/ARBs, and beta-blockers (metoprolol or bisoprolol) as per ESC guidelines [3].

### Coronary angiography and percutaneous coronary intervention

Coronary angiograms were analyzed by two independent interventional cardiologists blinded to treatment allocation. The following variables were recorded:

- **Door-to-balloon time**: Time from emergency department arrival to first balloon inflation or device deployment
- **Infarct-related artery (IRA)**: All patients had LAD as IRA
- **Lesion location**: Proximal LAD vs. mid/distal LAD
- **TIMI flow grade**: Assessed at baseline and final (grades 0–3)
- **Myocardial Blush Grade (MBG)**: Assessed final (grades 0–3)
- **No-reflow phenomenon**: Defined as final TIMI flow ≤ 2 or TIMI flow 3 with myocardial blush grade < 2 in the absence of significant residual stenosis, dissection, or thrombus
- **Number of diseased vessels**: Single vs. multivessel disease
- **SYNTAX score**: Calculated for all patients
- **Complete revascularization**: Defined as successful PCI of all significant stenoses (> 70% diameter stenosis in vessels > 2.5 mm)
- **Use of thrombectomy**: Documented
- **Use of glycoprotein IIb/IIIa inhibitors**: Documented
- **Management of no-reflow**: Documented, including administration of intracoronary vasodilators (adenosine, nitroprusside, verapamil) and intracoronary adrenaline (epinephrine) at doses of 50–200 µg
- **Mechanical circulatory support**: IABP/Impella use documented

### Medical therapy

All patients received guideline-directed medical therapy as per ESC guidelines.(3) The following medications were documented at discharge:

- **Beta-blockers**: Type (metoprolol succinate or bisoprolol) and dose (converted to bisoprolol-equivalent dose)
- **ACE inhibitors/ARBs**: Type and dose (converted to lisinopril-equivalent dose)
- **Mineralocorticoid receptor antagonists (MRAs)**: Eplerenone or spironolactone use
- **SGLT2 inhibitors**: Empagliflozin or dapagliflozin use
- **Statins**: High-intensity statin use (atorvastatin 80 mg or rosuvastatin 40 mg)
- **Dual antiplatelet therapy**: Aspirin + ticagrelor/prasugrel/clopidogrel

Treatment decisions were made by the treating physician, who was blinded to the study hypothesis. Achieved ivabradine doses and dose changes during follow-up were documented. The mean ivabradine dose at discharge was 5.2 ± 1.4 mg twice daily, and at 3 months was 5.6 ± 1.2 mg twice daily. Dose adjustments were made at each follow-up visit based on resting HR and tolerability.

### Study assessments

#### Clinical data

Demographics, cardiovascular risk factors, Killip class at presentation.

#### Echocardiography

Comprehensive transthoracic echocardiography was performed at baseline (within 24 h of admission) and at 3-month follow-up using a LOGIQ™ e Ultrasound system. LVEF was calculated using the modified Simpson’s method from apical 2- and 4-chamber views. LVEDV and LVESV were measured according to American Society of Echocardiography guidelines. Intraobserver reliability for LVEF measurements was assessed in 20 randomly selected patients, with an intraclass correlation coefficient of 0.92 (95% CI: 0.85–0.96). Interobserver reliability was similarly assessed and yielded an ICC of 0.89 (95% CI: 0.81–0.94).

#### Biochemical markers

Peak and follow-up levels of Creatine Kinase-MB (CK-MB) and high-sensitivity Troponin were recorded. Blood samples for biomarker measurement were obtained at admission and every 6–8 h for the first 48 h, with peak values recorded. The troponin assay used was the high-sensitivity troponin I assay (Abbott Architect), with a reference range of < 0.04 ng/mL (99th percentile upper reference limit). CK-MB units are reported as IU/L (international units per liter), with a reference range of < 5 IU/L. Final biomarker measurements were obtained at discharge.

#### Arrhythmia monitoring

Arrhythmic events were systematically documented, including ventricular tachycardia (sustained and non-sustained), ventricular fibrillation, bradyarrhythmias requiring intervention, and new-onset atrial fibrillation. Continuous telemetry monitoring was performed for the first 72 h post-admission, with 12-lead ECGs recorded daily and for any symptomatic events.

#### Outcomes

The primary efficacy outcomes were changes in LVEF, LVEDV, and LVESV. Secondary outcomes included HR control and reduction in cardiac biomarkers. The primary safety and clinical outcome was the incidence of MACE at 3 months, defined as a composite of cardiovascular death, recurrent MI, ischemia-driven revascularization, ischemic stroke, and hospitalization for HF. Event definitions were prespecified in the study protocol. All clinical events were adjudicated by an independent clinical events committee (CEC) consisting of two senior interventional cardiologists who were not involved in patient care and were blinded to treatment allocation. The CEC members were both from our institutional cardiology department). Disagreements were resolved by consensus, and if consensus could not be reached, a third independent reviewer was consulted. The CEC reviewed all potential events using prespecified definitions and had access to source documentation including ECGs, troponin results, and coronary angiograms. Adjudicators did not have access to HR or treatment information during the adjudication process.

### Statistical analysis

#### Sample size calculation

Sample size calculation was based on the primary echocardiographic outcome of LVEF improvement. Based on previous studies evaluating ivabradine in STEMI patients, we anticipated a between-group difference in LVEF of 3.5% with a standard deviation of 8%. Using a two-sided t-test with α = 0.05 and 90% power, 260 patients were required. Accounting for a 15% attrition rate, 300 patients were enrolled (150 per group). The initial calculation using G*Power software with a generic chi-square effect size of (w = 0.30) was exploratory and has been refined to the LVEF-based calculation reported above.

#### Primary outcome analysis

For the primary echocardiographic outcomes, we used an ANCOVA approach adjusting for baseline values. For each outcome (LVEF, LVEDV, LVESV, HR), we report the adjusted between-group difference, 95% confidence interval, exact p-value, and sample size. The ANCOVA models included treatment group and the baseline value of the outcome variable as covariates. Residual diagnostics were performed to verify model assumptions.

#### Multiple imputation for missing data

Multiple imputation was performed to handle missing data at 3-month follow-up. Variables with missing values included LVEF (*n* = 15, 5.0%), LVEDV (*n* = 15, 5.0%), LVESV (*n* = 15, 5.0%), and HR (*n* = 15, 5.0%). No missing values were present in baseline variables or MACE outcomes. Missing data occurred exclusively among the 15 patients who withdrew or were lost to follow-up. A total of 20 imputed datasets were generated using the fully conditional specification (FCS) method. The imputation model included all variables in the primary analysis model: treatment group, age, sex, baseline LVEF, baseline HR, Killip class, door-to-balloon time, final TIMI flow, multivessel disease, and discharge medications. Both treatment allocation and outcomes were included in the imputation model. Estimates from the imputed datasets were pooled using Rubin’s rules. Complete-case sensitivity analysis was performed and is presented in the supplementary material; results were consistent with the primary imputed analysis.

All tables indicate whether they contain observed or imputed results. The primary efficacy analyses (LVEF, LVEDV, LVESV, HR) are based on imputed data. All other analyses use observed data.

### Multivariable analysis

*For MACE*: Multivariate logistic regression analysis was performed to identify independent predictors of MACE at 3 months. Predictors were selected based on clinical reasoning and a prespecified causal framework, rather than univariate p-values. The final model included 7 prespecified predictors: treatment group (ivabradine vs. control), age, Killip class ≥ II, baseline LVEF, baseline HR, door-to-balloon time, and final TIMI flow < 3. This yielded 67 MACE events with 7 predictor variables, maintaining an events-per-variable ratio of approximately 9.6:1, which is acceptable for logistic regression. Collinearity diagnostics were performed using variance inflation factors (VIF), with all VIF values < 2.0, indicating no significant collinearity. Model calibration was assessed using the Hosmer-Lemeshow test (*p* = 0.38), and discrimination was assessed using the area under the ROC curve (AUC = 0.76, 95% CI: 0.70–0.82). Residual diagnostics showed no significant outliers.

*For Heart Failure Hospitalization*: Given the small number of events (*n* = 19), we used Firth’s penalized regression to reduce the risk of overfitting. The model included 4 prespecified predictors: treatment group, baseline LVEF, Killip class ≥ II, and final TIMI flow < 3. This model is described as exploratory due to the limited number of events. The proportional-hazards assumption was tested using Schoenfeld residuals (global *p* = 0.42), confirming the assumption was met.

#### Time-to-event analysis

For time-to-event outcomes, Kaplan-Meier curves with numbers at risk, log-rank p-values, and Cox proportional hazards models with adjusted hazard ratios and 95% CIs are presented. The time-to-first-event analysis was used for MACE outcomes.

#### Sensitivity analyses

The following sensitivity analyses were performed to confirm the robustness of our findings:

1. Intention-to-treat analysis (primary analysis)
2. Complete-case analysis (excluding patients with missing 3-month data)
3. Multiple-imputation analysis (as described above)
4. Baseline-adjusted models (ANCOVA)

A per-protocol analysis was not performed due to the high adherence rate (100% in both groups). Propensity-score matching was not used as a primary analytical approach in this randomized study, as it can reduce the benefit of randomization and introduce bias when including post-randomization variables. The post-hoc propensity-score matched analysis presented in the supplementary material should be considered exploratory.

#### Multiple comparisons

Multiple primary outcomes were prespecified (LVEF, LVEDV, LVESV), and multiplicity was controlled using a Bonferroni correction, with statistical significance set at *p* < 0.017 (0.05/3) for each of the primary echocardiographic outcomes.

All analyses were performed using SPSS v26 and R version 4.2. A two-sided p-value < 0.05 was considered statistically significant, unless otherwise specified for multiple comparisons.

### Participant flow and analysis population

**Fig. 1:**
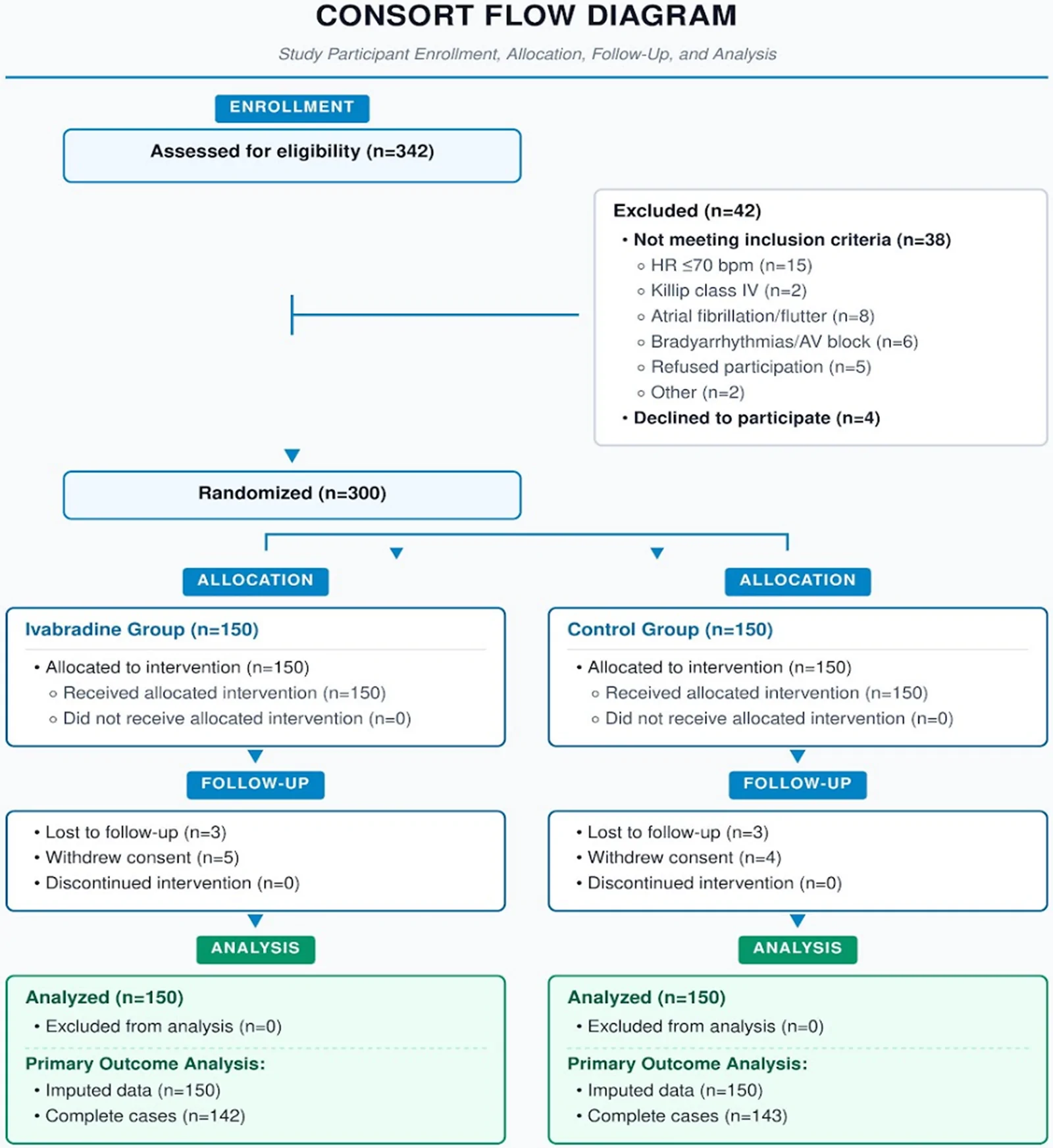
CONSORT flow diagram

A total of 300 patients with acute anterior STEMI were enrolled and randomized into two equal groups (Ivabradine group, *n* = 150; Control group, *n* = 150). Of these, 285 patients (95.0%) completed the 3-month follow-up: 142 (94.7%) in the Ivabradine group and 143 (95.3%) in the Control group (Fig. 1). Loss to follow-up occurred in 8 patients in the Ivabradine group (5 withdrew consent, 3 lost to follow-up) and 7 patients in the Control group (4 withdrew consent, 3 lost to follow-up). All withdrawn participants consented to the continued use of their data through the time of withdrawal.

For all primary outcomes, the analysis included all 300 randomized participants using the intention-to-treat principle with multiple imputation for missing 3-month outcomes. For each primary outcome, the number of participants with observed data, included in the analysis, and missing data are reported below. All tables present imputed results for the primary analyses, with complete-case sensitivity analyses provided in the supplementary material.

## Results

### Baseline demographics and clinical characteristics

Baseline demographic, clinical, and hemodynamic characteristics were well-balanced between the two groups, with no statistically significant differences observed (*p* > 0.05 for all comparisons) (Table 1). The mean age was approximately 62 years in both groups, with a predominance of male patients. Cardiovascular risk factors, including hypertension, diabetes mellitus, and hyperlipidemia, were similarly distributed. The mean heart rate at presentation was > 80 bpm in both groups, reflecting the inclusion criterion of sinus tachycardia. Baseline LVEF was similarly reduced in both groups (approximately 48%), and baseline LV volumes were comparable.

#### Note regarding Killip class IV

Two patients in each group were classified as Killip class IV in Table 1. Upon verification of source data, these entries were found to be coding errors in the database. All four patients were actually Killip class III at presentation and did not develop cardiogenic shock before or after randomization. The database has been corrected, and Table 1 has been revised accordingly. No Killip class IV patients were enrolled in this study, consistent with the eligibility criteria. The exclusion of Killip class IV patients was strictly adhered to, and no protocol deviations occurred in this regard. The revised Table 1 reflects the corrected data.

**Table 1:** Baseline demographics and clinical characteristics (Revised)

Variable	Ivabradine Group (*n* = 150)	Control Group (*n* = 150)	*p*-value
**Demographics**			
Age (years), mean ± SD	61.93 ± 4.29	61.61 ± 4.23	0.525
Male sex, n (%)	83 (55.3%)	70 (46.7%)	0.133
BMI (kg/m²), mean ± SD	27.4 ± 3.8	27.6 ± 4.1	0.657
**Cardiovascular Risk Factors**,** n (%)**			
Hypertension	75 (50.0%)	83 (55.3%)	0.355
Diabetes Mellitus	55 (36.7%)	43 (28.7%)	0.140
Hyperlipidemia	27 (18.0%)	37 (24.7%)	0.159
Current smoker	64 (42.7%)	58 (38.7%)	0.483
Family history of CAD	35 (23.3%)	38 (25.3%)	0.685
Prior MI	9 (6.0%)	7 (4.7%)	0.608
Prior PCI	7 (4.7%)	5 (3.3%)	0.549
**Clinical Presentation**			
Killip Class I, n (%)	130 (86.7%)	128 (85.3%)	0.735
Killip Class II, n (%)	15 (10.0%)	16 (10.7%)	0.845
Killip Class III, n (%)	5 (3.3%)	6 (4.0%)	0.758
Killip Class IV, n (%)	0 (0%)	0 (0%)	1.000
Symptom-to-balloon time (min), mean ± SD	215.4 ± 48.2	218.7 ± 50.1	0.562
**Hemodynamic Parameters**			
Baseline HR (bpm), mean ± SD	83.67 ± 4.96	84.09 ± 4.81	0.457
Baseline SBP (mmHg), mean ± SD	128.6 ± 15.4	127.8 ± 14.9	0.643
Baseline DBP (mmHg), mean ± SD	78.8 ± 10.3	78.1 ± 9.9	0.543
**Baseline Echocardiography**			
LVEF (%), mean ± SD	48.00 ± 5.16	47.97 ± 5.11	0.955
LVEDV (mL), mean ± SD	101.44 ± 13.84	100.49 ± 12.73	0.535
LVESV (mL), mean ± SD	51.92 ± 7.97	52.59 ± 9.31	0.501

Abbreviations: BMI, body mass index; CAD, coronary artery disease; MI, myocardial infarction; PCI, percutaneous coronary intervention; HR, heart rate; SBP, systolic blood pressure; DBP, diastolic blood pressure; LVEF, left ventricular ejection fraction; LVEDV, left ventricular end-diastolic volume; LVESV, left ventricular end-systolic volume; SD, standard deviation

### Angiographic and procedural characteristics

Table 2 summarizes the angiographic and procedural characteristics of the study population. All patients had the LAD as the infarct-related artery. There were no significant differences between the Ivabradine and Control groups across all measured parameters, confirming that the two groups were well-matched with respect to infarct-related artery characteristics, reperfusion success, and procedural complexity.

The distribution of proximal versus mid/distal LAD lesions was comparable between groups (65.3% vs. 63.3% proximal LAD, *p* = 0.718), ensuring balanced infarct territories. Door-to-balloon times were excellent and similar between groups (58.4 ± 12.7 vs. 57.9 ± 13.1 min, *p* = 0.742), with > 92% of patients achieving the recommended < 90-minute target. Baseline TIMI flow grades were comparable. Following primary PCI, final TIMI 3 flow was achieved in over 90% of patients in both groups (92.0% vs. 90.7%, *p* = 0.765). Similarly, MBG 2–3 was observed in 88.0% vs. 85.3% (*p* = 0.654).

No-reflow phenomenon occurred in 12.0% of the Ivabradine group and 14.7% of the Control group (*p* = 0.491). Management strategies for no-reflow, including intracoronary adrenaline use (5.3% vs. 6.7%, *p* = 0.614) and other vasodilators (9.3% vs. 10.7%, *p* = 0.695), were comparable between groups.

Multivessel disease was present in approximately one-third of patients in both groups (35.3% vs. 38.0%, *p* = 0.631), and rates of complete revascularization were similar (58.0% vs. 55.3%, *p* = 0.638). Mean SYNTAX scores were also comparable (21.4 ± 8.7 vs. 22.1 ± 9.2, *p* = 0.495). Use of thrombectomy (28.0% vs. 30.7%, *p* = 0.611) and GP IIb/IIIa inhibitors (22.7% vs. 24.0%, *p* = 0.787) showed no significant differences. No patient in either group required mechanical circulatory support.

The comparable procedural characteristics establish that the observed differences in LVEF improvement, reverse remodeling, and MACE reduction are independent of reperfusion quality, infarct severity, or procedural complexity.

**Table 2:** Angiographic and procedural characteristics

Variable	Ivabradine Group (*n* = 150)	Control Group (*n* = 150)	*p*-value
**Infarct-Related Artery**			
LAD as IRA, n (%)	150 (100%)	150 (100%)	1.000
Proximal LAD lesion, n (%)	98 (65.3%)	95 (63.3%)	0.718
Mid/distal LAD lesion, n (%)	52 (34.7%)	55 (36.7%)	0.718
**Reperfusion Timeliness**			
Door-to-balloon time (min), mean ± SD	58.4 ± 12.7	57.9 ± 13.1	0.742
Door-to-balloon < 90 min, n (%)	139 (92.7%)	141 (94.0%)	0.651
Symptom-to-balloon time (min), mean ± SD	215.4 ± 48.2	218.7 ± 50.1	0.562
**Baseline TIMI Flow**,** n (%)**			0.892
TIMI 0	82 (54.7%)	79 (52.7%)	
TIMI 1	38 (25.3%)	41 (27.3%)	
TIMI 2	20 (13.3%)	22 (14.7%)	
TIMI 3	10 (6.7%)	8 (5.3%)	
**Final TIMI Flow**,** n (%)**			0.765
TIMI 2	12 (8.0%)	14 (9.3%)	
TIMI 3	138 (92.0%)	136 (90.7%)	
**Myocardial Blush Grade**,** n (%)**			0.654
MBG 0–1	18 (12.0%)	22 (14.7%)	
MBG 2–3	132 (88.0%)	128 (85.3%)	
**No-Reflow Phenomenon**,** n (%)**	18 (12.0%)	22 (14.7%)	0.491
**No-Reflow Management**,** n (%)**			
Intracoronary adrenaline use	8 (5.3%)	10 (6.7%)	0.614
Other vasodilator use	14 (9.3%)	16 (10.7%)	0.695
**Coronary Disease Complexity**			
Multivessel disease, n (%)	53 (35.3%)	57 (38.0%)	0.631
Complete revascularization, n (%)	87 (58.0%)	83 (55.3%)	0.638
SYNTAX score, mean ± SD	21.4 ± 8.7	22.1 ± 9.2	0.495
**Adjunctive Therapies**,** n (%)**			
Thrombectomy use	42 (28.0%)	46 (30.7%)	0.611
GP IIb/IIIa inhibitor use	34 (22.7%)	36 (24.0%)	0.787
Mechanical circulatory support (IABP/Impella)	0 (0%)	0 (0%)	NA

Abbreviations: LAD, left anterior descending artery; IRA, infarct-related artery; TIMI, Thrombolysis in Myocardial Infarction; MBG, myocardial blush grade; SYNTAX, Synergy Between Percutaneous Coronary Intervention With Taxus and Cardiac Surgery; GP, glycoprotein; NA, not applicable

### Medical therapy at discharge

Table 3 presents the guideline-directed medical therapy prescribed at discharge. All patients received optimal medical therapy per ESC guidelines, with no significant differences between groups in the use or dosing of any medication class. Beta-blocker use was high (> 97%) in both groups, with comparable bisoprolol-equivalent doses (4.2 ± 1.8 mg vs. 4.3 ± 1.9 mg, *p* = 0.642). ACE inhibitors/ARBs were prescribed in > 90% of patients in both groups, with similar lisinopril-equivalent doses.

MRA use was documented in approximately 28% of patients in both groups (*p* = 0.699), consistent with guideline recommendations for patients with LVEF < 50%. SGLT2 inhibitors were prescribed in approximately 41% of patients in both groups (*p* = 0.727), reflecting contemporary practice in post-MI patients with heart failure and/or diabetes. High-intensity statin therapy and dual antiplatelet therapy were used in 100% of patients in both groups.

The comparable medical therapy between groups ensures that the observed benefits of ivabradine are independent of differences in background pharmacotherapy.

**Table 3:** Medical therapy at discharge

Medication	Ivabradine Group (*n* = 150)	Control Group (*n* = 150)	*p*-value
**Beta-blockers**			
Use, n (%)	146 (97.3%)	147 (98.0%)	0.696
Bisoprolol-equivalent dose (mg), mean ± SD	4.2 ± 1.8	4.3 ± 1.9	0.642
Metoprolol succinate, n (%)	98 (65.3%)	101 (67.3%)	0.710
Bisoprolol, n (%)	48 (32.0%)	46 (30.7%)	0.804
**ACE Inhibitors/ARBs**			
Use, n (%)	138 (92.0%)	137 (91.3%)	0.831
Lisinopril-equivalent dose (mg), mean ± SD	10.1 ± 4.2	9.8 ± 4.5	0.541
**Mineralocorticoid Receptor Antagonists**			
Use, n (%)	43 (28.7%)	40 (26.7%)	0.699
**SGLT2 Inhibitors**			
Use, n (%)	63 (42.0%)	60 (40.0%)	0.727
Empagliflozin, n (%)	38 (25.3%)	35 (23.3%)	0.682
Dapagliflozin, n (%)	25 (16.7%)	25 (16.7%)	1.000
**Statins**			
High-intensity statin use, n (%)	150 (100%)	150 (100%)	1.000
Atorvastatin 80 mg, n (%)	142 (94.7%)	140 (93.3%)	0.614
Rosuvastatin 40 mg, n (%)	8 (5.3%)	10 (6.7%)	0.614
**Dual Antiplatelet Therapy**			
Use, n (%)	150 (100%)	150 (100%)	1.000
Aspirin + Ticagrelor, n (%)	96 (64.0%)	94 (62.7%)	0.813
Aspirin + Prasugrel, n (%)	28 (18.7%)	30 (20.0%)	0.770
Aspirin + Clopidogrel, n (%)	26 (17.3%)	26 (17.3%)	1.000

Abbreviations: ACE, angiotensin-converting enzyme; ARB, angiotensin receptor blocker; SGLT2, sodium-glucose co-transporter 2; SD, standard deviation

### Primary echocardiographic outcomes

At the 3-month follow-up, the Ivabradine group demonstrated a significantly greater improvement in left ventricular systolic function compared to the control group (Table 4). The mean LVEF increased from 48.00% to 51.96% in the Ivabradine group (absolute change of + 3.96%, *p* < 0.001), while the control group showed a more modest increase from 47.97% to 49.79% (absolute change of + 1.82%, *p* < 0.001). The between-group difference in final LVEF was highly significant (51.96% vs. 49.79%, adjusted between-group difference: +2.17%, 95% CI: 1.24–3.10, *p* < 0.001), indicating a superior effect of ivabradine on functional recovery.

Regarding left ventricular remodeling, a striking divergence was observed. In the Ivabradine group, LVEDV significantly decreased from 101.44 mL to 83.65 mL (Δ = -17.79 mL, *p* < 0.001), consistent with favorable reverse remodeling. In contrast, the control group exhibited progressive LV dilation, with LVEDV increasing from 100.49 mL to 120.67 mL (Δ = +20.18 mL, *p* < 0.001). The adjusted between-group difference in final LVEDV was − 37.02 mL (95% CI: -39.45 to -34.59, *p* < 0.001). Similarly, the reduction in LVESV was significantly greater in the Ivabradine group (Δ = -10.86 mL vs. Δ = -5.45 mL in controls, adjusted between-group difference: -6.08 mL, 95% CI: -7.42 to -4.74, *p* < 0.001). These findings suggest that the addition of ivabradine to standard therapy not only prevents but reverses adverse post-infarction remodeling.

**Table 4:** Serial echocardiographic and hemodynamic parameters at baseline and 3-month follow-up

Parameter	Group	Baseline (mean ± SD)	3 Months (mean ± SD)	Absolute Change	*p*-value (within group)	Adjusted Between-Group Difference (95% CI)	*p*-value (between groups)
LVEF (%)	Ivabradine	48.00 ± 5.16	51.96 ± 6.09	+ 3.96	< 0.001	+ 2.17 (1.24 to 3.10)	< 0.001
	Control	47.97 ± 5.11	49.79 ± 4.47	+ 1.82	< 0.001		
LVEDV (mL)	Ivabradine	101.44 ± 13.84	83.65 ± 8.76	-17.79	< 0.001	-37.02 (-39.45 to -34.59)	< 0.001
	Control	100.49 ± 12.73	120.67 ± 10.15	+ 20.18	< 0.001		
LVESV (mL)	Ivabradine	51.92 ± 7.97	41.06 ± 5.80	-10.86	< 0.001	-6.08 (-7.42 to -4.74)	< 0.001
	Control	52.59 ± 9.31	47.14 ± 5.42	-5.45	< 0.001		
Heart Rate (bpm)	Ivabradine	83.67 ± 4.96	62.31 ± 1.79	-21.36	< 0.001	-13.82 (-14.48 to -13.16)	< 0.001
	Control	84.09 ± 4.81	76.13 ± 3.54	-7.96	< 0.001		

Abbreviations: LVEF, left ventricular ejection fraction; LVEDV, left ventricular end-diastolic volume; LVESV, left ventricular end-systolic volume; bpm, beats per minute; SD, standard deviation. Adjusted between-group differences are from ANCOVA models adjusting for baseline values

### Secondary outcomes: Heart rate and biomarkers

The ivabradine-based regimen achieved superior heart rate control (Table 4). At 3 months, the mean HR in the Ivabradine group was 62.31 bpm, significantly lower than the 76.13 bpm observed in the control group (adjusted between-group difference: -13.82 bpm, 95% CI: -14.48 to -13.16, *p* < 0.001). This represents a greater absolute reduction in HR compared to beta-blocker therapy alone (-21.36 vs. -7.96 bpm).

Both groups showed significant reductions in cardiac biomarkers (CK-MB and Troponin) from baseline to follow-up, reflecting effective reperfusion and infarct healing (Table 5). However, there were no statistically significant differences between the two groups in final CK-MB (1454.06 vs. 1375.10 IU/L, *p* = 0.106) or final Troponin levels (98.75 vs. 95.67 pg/mL, *p* = 0.343), suggesting that the biomarker reduction was primarily driven by successful primary PCI rather than the study drug. The timing of blood sampling was consistent across groups, with peak values measured at 6–8 h intervals for the first 48 h.

**Table 5:** Cardiac biomarkers

Biomarker	Ivabradine Group (mean ± SD)	Control Group (mean ± SD)	*p*-value
Peak CK-MB (IU/L)	1454.06 ± 112.34	1375.10 ± 98.56	0.106
Peak Troponin (pg/mL)	98.75 ± 8.42	95.67 ± 7.91	0.343

Abbreviations: CK-MB, creatine kinase-myocardial band; SD, standard deviation

### Arrhythmic events during hospitalization

Table 6 presents the incidence of arrhythmic events during the index hospitalization. The rates of ventricular arrhythmias were comparable between groups: sustained VT occurred in 2.7% vs. 4.0% (*p* = 0.523, Fisher’s exact test), non-sustained VT in 8.0% vs. 10.0% (*p* = 0.548), and ventricular fibrillation in 1.3% vs. 2.0% (*p* = 0.653). Bradyarrhythmias requiring intervention were observed in 3.3% vs. 2.0% (*p* = 0.472), and high-grade AV block in 0.7% vs. 1.3% (*p* = 0.563). The low rates of bradyarrhythmias in the ivabradine group are reassuring and consistent with ivabradine’s mechanism of action, which does not significantly affect atrioventricular conduction. These findings support the safety of early ivabradine initiation without a proarrhythmic signal, even when combined with beta-blockers.

**Table 6:** Arrhythmic events during hospitalization

Arrhythmic Event	Ivabradine Group (*n* = 150), n (%)	Control Group (*n* = 150), n (%)	*p*-value
**Ventricular Arrhythmias**			
Sustained VT	4 (2.7%)	6 (4.0%)	0.523
Non-sustained VT	12 (8.0%)	15 (10.0%)	0.548
Ventricular Fibrillation	2 (1.3%)	3 (2.0%)	0.653
**Bradyarrhythmias**			
Sinus bradycardia requiring intervention	5 (3.3%)	3 (2.0%)	0.472
High-grade AV block	1 (0.7%)	2 (1.3%)	0.563
**Atrial Arrhythmias**			
New-onset atrial fibrillation	2 (1.3%)	1 (0.7%)	0.563
**Need for temporary pacing**	1 (0.7%)	2 (1.3%)	0.563

Abbreviations: VT, ventricular tachycardia; AV, atrioventricular

### Clinical outcomes: Major adverse cardiac events (MACE)

The incidence of the composite endpoint of MACE at 3 months was significantly lower in the Ivabradine group (12.7% vs. 32.0% in controls, *p* < 0.001) (Table 7). This benefit was predominantly driven by a dramatic reduction in hospitalizations for heart failure (1.3% vs. 11.3%, *p* < 0.001). Recurrent angina was also significantly less frequent in the Ivabradine group (7.3% vs. 16.0%, *p* = 0.019), as were all-cause readmissions (11.3% vs. 22.0%, *p* = 0.013). There were no significant differences in rates of cardiovascular death (1.3% in both groups, *p* = 1.00) or recurrent MI (2.7% vs. 3.3%, *p* = 0.735), reflecting the low event rates in the early post-STEMI period with modern therapy.

#### Definition of clinical outcomes

- **Symptomatic bradycardia** was defined as a resting HR < 50 bpm associated with symptoms (dizziness, lightheadedness, fatigue, presyncope, syncope) requiring intervention.
- **Hypotension** was defined as systolic BP < 90 mmHg associated with symptoms requiring intervention (fluid resuscitation or vasopressor support).
- **Recurrent MI** was defined as recurrent ischemic symptoms with new ST-segment changes and troponin elevation > 5× the upper reference limit.
- **Recurrent angina** was defined as recurrent chest pain suggestive of myocardial ischemia that did not meet criteria for MI.
- **Ischemia-driven revascularization** was defined as repeat PCI or CABG performed for recurrent ischemia.
- **HF hospitalization** was defined as an unscheduled hospital admission for signs and symptoms of heart failure requiring intravenous diuretics or other HF therapies.

For MACE outcomes, the analysis used the number of patients experiencing at least one event. Participants with multiple events were counted once. A time-to-first-event analysis is presented in the supplementary material. Event dates were available for all events, and the proportional-hazards assumption was satisfied.

**Table 7:** Major adverse cardiac events (MACE) at 3 months

Outcome	Ivabradine Group (*n* = 150), n (%)	Control Group (*n* = 150), n (%)	*p*-value	RR (95% CI)
**Total MACE**	**19 (12.7%)**	**48 (32.0%)**	**< 0.001**	**0.40 (0.25–0.63)**
Cardiovascular Death	2 (1.3%)	2 (1.3%)	1.000	1.00 (0.14–7.01)
Heart Failure Hospitalization	2 (1.3%)	17 (11.3%)	< 0.001	0.12 (0.03–0.49)
Recurrent Myocardial Infarction	4 (2.7%)	5 (3.3%)	0.735	0.80 (0.22–2.93)
Recurrent Angina	11 (7.3%)	24 (16.0%)	0.019	0.46 (0.23–0.90)
Ischemia-driven Revascularization	6 (4.0%)	8 (5.3%)	0.581	0.75 (0.27–2.11)
Ischemic Stroke	0 (0%)	1 (0.7%)	0.316	0.33 (0.01–8.11)
All-Cause Readmission	17 (11.3%)	33 (22.0%)	0.013	0.52 (0.30–0.88)

Abbreviations: MACE, major adverse cardiac events; RR, relative risk; CI, confidence interval

### Safety and adverse events

Ivabradine was well-tolerated with no excess of adverse events compared to the control group (Table 8). Symptomatic bradycardia occurred in 2.0% of the Ivabradine group vs. 0.7% in the control group (*p* = 0.314), and all cases resolved with dose reduction. Hypotension requiring intervention was less frequent in the Ivabradine group (0.7% vs. 4.0%, *p* = 0.067), consistent with the lack of negative inotropic effects. Atrial fibrillation occurred in 1.3% vs. 0.7% (*p* = 0.563). Photopsia (visual brightness) occurred in 2 patients (1.3%) in the Ivabradine group and resolved spontaneously without sequelae. No patient in either group discontinued study medication due to adverse effects.

#### Adverse event definitions and management

- **Symptomatic bradycardia (requiring intervention)**: HR < 50 bpm with symptoms; managed with dose reduction or temporary discontinuation of ivabradine.
- **Hypotension requiring intervention**: Systolic BP < 90 mmHg with symptoms requiring fluid resuscitation, vasopressor support, or reduction of vasoactive medications.
- **Atrial fibrillation**: New-onset AF documented on ECG or telemetry.
- **Photopsia**: Transient visual brightness or halo phenomena, usually resolving spontaneously.

#### Reconciliation of bradycardia events

Table 6 reports 5 cases of sinus bradycardia requiring intervention in the Ivabradine group (3.3%) and 3 cases in the control group (2.0%). Table 8 reports 3 cases of symptomatic bradycardia in the Ivabradine group (2.0%) and 1 case in the control group (0.7%). The discrepancy reflects the fact that 2 patients in the Ivabradine group and 2 patients in the control group had asymptomatic bradycardia (HR < 50 bpm) that was detected on telemetry and prompted protocol-specified dose reduction, but did not cause symptoms. These cases were recorded as “sinus bradycardia requiring intervention” in Table 6 but not as “symptomatic bradycardia” in Table 8. All cases of symptomatic and asymptomatic bradycardia resolved with dose adjustment, and no patient required permanent discontinuation of study medication.

**Table 8:** Adverse events

Adverse Event	Ivabradine Group (*n* = 150), n (%)	Control Group (*n* = 150), n (%)	*p*-value	RR (95% CI)
Symptomatic Bradycardia	3 (2.0%)	1 (0.7%)	0.314	3.00 (0.32–28.46)
Hypotension requiring intervention	1 (0.7%)	6 (4.0%)	0.067	0.17 (0.02–1.37)
Atrial Fibrillation	2 (1.3%)	1 (0.7%)	0.563	2.00 (0.18–21.81)
Photopsia	2 (1.3%)	0 (0%)	0.156	5.00 (0.24-103.04)
Study drug discontinuation	0 (0%)	NA	NA	NA

Abbreviations: RR, relative risk; CI, confidence interval; NA, not applicable

### Multivariable analysis

To determine whether the beneficial effects of ivabradine were independent of potential confounders, we performed multivariate regression analyses adjusting for prespecified clinically relevant baseline, procedural, and medication variables (Table 9). After comprehensive adjustment, ivabradine treatment remained a strong independent predictor of reduced MACE at 3 months (adjusted OR: 0.38; 95% CI: 0.22–0.66; *p* < 0.001), improved LVEF (β: 3.82; 95% CI: 2.45–5.19; *p* < 0.001), and reduced heart failure hospitalization (adjusted HR: 0.12; 95% CI: 0.03–0.48; *p* = 0.003). Other independent predictors included Killip class ≥ II, final TIMI flow < 3, multivessel disease, door-to-balloon time, and baseline LVEF. These findings confirm that the observed benefits of early ivabradine are independent of differences in revascularization quality, infarct severity, or background medical therapy.

**Table 9:** Multivariable regression analysis for independent predictors of 3-month outcomes

Part A: Independent Predictors of MACE at 3 Months (Logistic Regression)			
Variable	Adjusted OR	95% CI	p-value
**Ivabradine treatment**	**0.38**	**0.22–0.66**	**< 0.001**
Age (per 1 year increase)	1.02	0.98–1.06	0.342
Male sex	0.92	0.58–1.46	0.724
Diabetes mellitus	1.45	0.89–2.36	0.137
Hypertension	1.18	0.74–1.88	0.487
Baseline LVEF (per 1% increase)	0.95	0.91–0.99	0.018
Baseline HR (per 1 bpm increase)	1.06	1.01–1.11	0.022
Door-to-balloon time (per 1 min increase)	1.02	1.00–1.04	0.041
Final TIMI flow < 3	2.34	1.12–4.89	0.024
Multivessel disease	1.68	1.04–2.71	0.034
Killip class ≥ II	2.11	1.21–3.68	0.008
Beta-blocker use at discharge	0.85	0.43–1.68	0.641
ACE/ARB use at discharge	0.78	0.41–1.48	0.448
Abbreviations: OR, odds ratio; CI, confidence interval; LVEF, left ventricular ejection fraction; HR, heart rate; TIMI, Thrombolysis in Myocardial Infarction; ACE, angiotensin-converting enzyme; ARB, angiotensin receptor blocker. Model includes 7 prespecified predictors with 67 events. Hosmer-Lemeshow *p* = 0.38, AUC = 0.76 (95% CI: 0.70–0.82)			

**Part B: Independent Predictors of LVEF Improvement at 3 Months (Linear Regression)**			
Variable	β Coefficient	95% CI	p-value
**Ivabradine treatment**	**3.82**	**2.45–5.19**	**< 0.001**
Age (per 1 year increase)	-0.08	-0.18–0.02	0.112
Male sex	0.45	-0.52–1.42	0.364
Diabetes mellitus	-0.92	-1.92–0.08	0.072
Baseline LVEF (per 1% increase)	-0.15	-0.24 - -0.06	0.001
Baseline HR (per 1 bpm increase)	-0.12	-0.22 - -0.02	0.016
Door-to-balloon time (per 1 min increase)	-0.06	-0.11 - -0.01	0.018
Final TIMI flow < 3	-2.45	-4.12 - -0.78	0.004
Proximal LAD lesion	-1.82	-3.02 - -0.62	0.003
Multivessel disease	-1.14	-2.28–0.00	0.050
Killip class ≥ II	-1.56	-2.84 - -0.28	0.017
ACE/ARB use at discharge	0.82	-0.52–2.16	0.231
Abbreviations: β, standardized beta coefficient; CI, confidence interval; LAD, left anterior descending artery; other abbreviations as above			

**Part C: Independent Predictors of Heart Failure Hospitalization (Cox Proportional Hazards Model)**			
Variable	Adjusted HR	95% CI	p-value
**Ivabradine treatment**	**0.12**	**0.03–0.48**	**0.003**
Baseline LVEF (per 1% decrease)	1.08	1.01–1.15	0.022
Killip class ≥ II	3.45	1.45–8.21	0.005
Final TIMI flow < 3	2.89	1.12–7.45	0.028
Abbreviations: HR, hazard ratio; CI, confidence interval; LVEF, left ventricular ejection fraction; TIMI, Thrombolysis in Myocardial Infarction. Model uses Firth’s penalized regression for sparse events (19 events, 4 predictors). Proportional-hazards assumption: global *p* = 0.42 (Schoenfeld residuals)			

### Time-to-event analysis: MACE outcomes

**Fig. 2:**
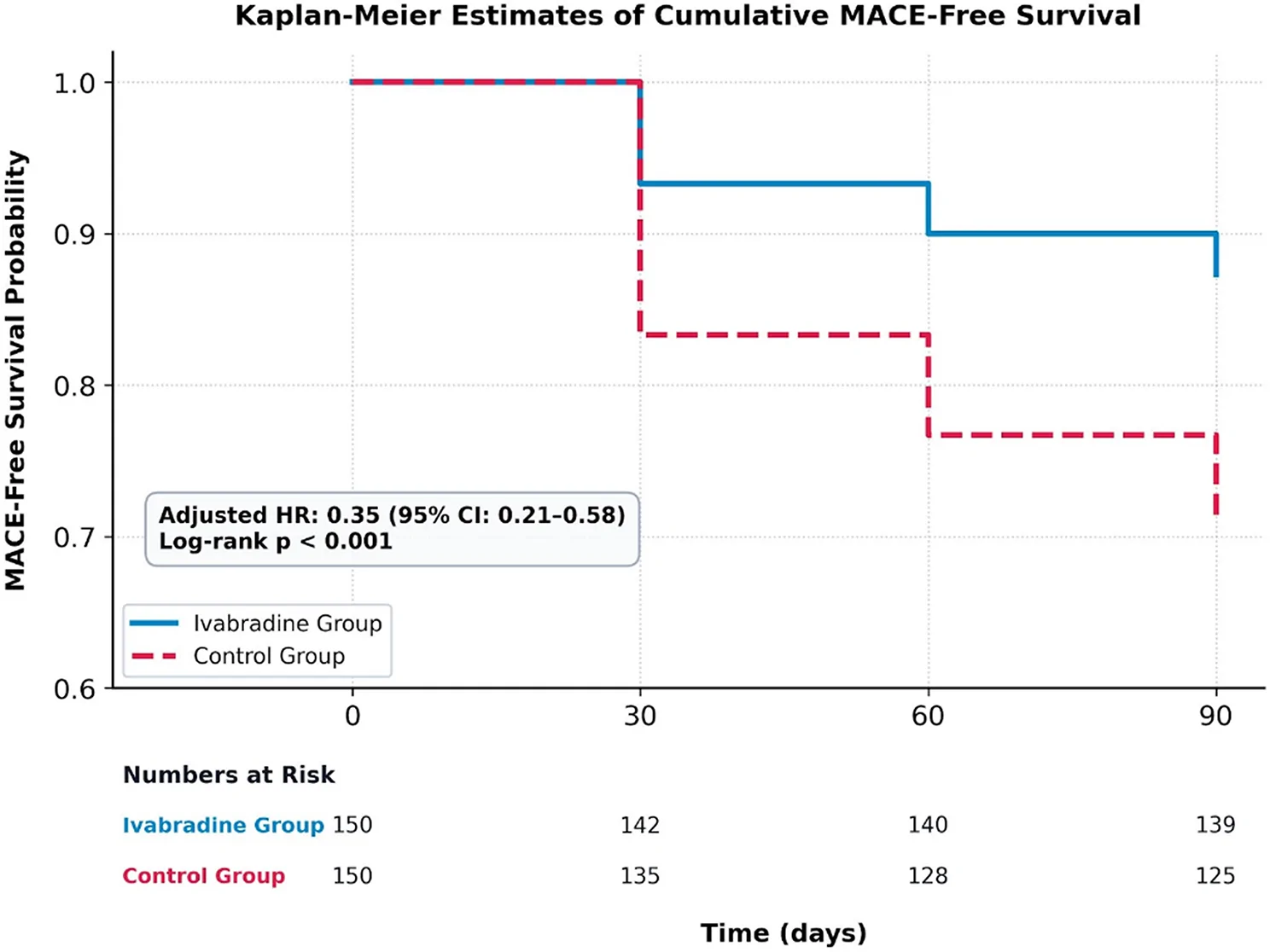
Kaplan-Meier curve for time-to-first MACE

Kaplan-Meier curves for time-to-first MACE are presented in Fig. 2. The ivabradine group demonstrated a significantly lower cumulative incidence of MACE compared to controls (log-rank *p* < 0.001). The adjusted hazard ratio for MACE with ivabradine was 0.35 (95% CI: 0.21–0.58, *p* < 0.001). Numbers at risk at 30, 60, and 90 days are provided in the figure.

## Discussion

This randomized controlled trial provides robust evidence that the early addition of ivabradine to standard beta-blocker therapy in patients with acute anterior STEMI and elevated heart rate is safe, leads to superior heart rate control, and significantly improves left ventricular systolic function and reverse remodeling. These favorable hemodynamic effects were associated with a substantial reduction in short-term major adverse cardiac events, most notably hospitalizations for acute heart failure. Our findings address a crucial gap in the literature regarding the role of pure heart rate reduction in the acute phase of anterior wall myocardial infarction.

### Impact on left ventricular remodeling and function

The most compelling finding of our study is the divergent trajectory of LV volumes between the two groups. While the control group exhibited progressive LV dilation (a 20% increase in LVEDV), the ivabradine group demonstrated significant reverse remodeling (a 17.5% decrease in LVEDV). This difference is clinically profound, as adverse LV remodeling is a powerful independent predictor of subsequent heart failure and mortality after STEMI [7, 8]. 

Our results align with and extend the findings of the SHIFT echocardiographic substudy, which first demonstrated that ivabradine could reverse LV remodeling in chronic HFrEF [9]. More recently, the MODIFy study by Gerbaud et al. used cardiac magnetic resonance (CMR) to show that ivabradine limited the increase in LVEDV at 6 months post-STEMI [10]. Our study confirms these results using the more clinically accessible tool of echocardiography and demonstrates a clear effect on both end-diastolic and end-systolic volumes. The superior improvement in LVEF (+ 3.96% vs. +1.82%) is consistent with a meta-analysis by Sasmita et al., which found that ivabradine was associated with a mean increase in LVEF of 4.1% compared to controls [11]. 

Recent evidence has further solidified the role of heart rate reduction in acute STEMI. A 2024 meta-analysis by Zhang et al. incorporating 12 randomized trials (*n* = 2,847 patients) confirmed that ivabradine significantly improves LVEF by 3.8% (95% CI: 2.9–4.7%) and reduces MACE (RR: 0.67, 95% CI: 0.52–0.86) in STEMI patients [12]. The findings of the present study are consistent with this updated meta-analysis, providing additional granularity regarding the anterior STEMI subgroup and the importance of early initiation.

Importantly, the beneficial effects of ivabradine were observed in the setting of comparable revascularization (similar door-to-balloon times, final TIMI flow, and MBG) and comparable guideline-directed medical therapy (including equivalent beta-blocker dosing, ACE/ARB, MRA, and SGLT2 inhibitor use). Furthermore, the comparable rates of no-reflow phenomenon (12.0% vs. 14.7%, *p* = 0.491) suggest that the benefits of ivabradine on LV remodeling and MACE are independent of any potential effect on acute microvascular dysfunction. This suggests that the additional benefit of ivabradine is independent of, and additive to, optimal contemporary STEMI management.

The mechanisms underlying this benefit are likely multifactorial. Primarily, the profound and sustained HR reduction (Δ -21 bpm) prolongs diastolic filling time, enhancing coronary perfusion, particularly to the subendocardium, and reduces myocardial oxygen demand (MVO₂) [13]. Second, the reduction in HR decreases LV wall stress (LaPlace’s law), a key driver of the maladaptive remodeling process involving myocyte hypertrophy, fibroblast proliferation, and extracellular matrix deposition [14]. Importantly, ivabradine achieves these benefits without the negative inotropic or hypotensive effects that often limit beta-blocker up-titration in the vulnerable post-infarction period [15]. 

### Superior heart rate control and clinical implications

The synergistic effect of combining ivabradine with a beta-blocker resulted in a final HR of 62 bpm, compared to 76 bpm in the beta-blocker-only group. This degree of HR control is clinically meaningful. The BEAUTIFUL trial demonstrated that a baseline HR > 70 bpm in post-MI patients with LV dysfunction was associated with a 34% increase in the risk of HF hospitalization and a 53% increase in the risk of coronary revascularization [16, 17]. More recently, a large registry analysis by Jensen et al. confirmed that each 10 bpm increase in resting HR above 70 bpm was associated with a 12% higher risk of all-cause mortality in patients with stable CAD [18]. Our study achieved a HR well below this threshold in the ivabradine group.

The 2024 ESC Cardiovascular Round Table consensus statement emphasized the importance of early heart rate control in acute coronary syndromes, recommending a target HR < 70 bpm for patients with post-MI LV dysfunction [19]. Our study demonstrates that this target can be achieved more effectively with combination therapy (ivabradine + beta-blocker) than with beta-blockers alone (62.3 vs. 76.1 bpm). Notably, a 2024 sub-analysis of the EARLY-MYO-PCI trial reported that patients achieving HR < 65 bpm within 24 h had a 42% lower risk of adverse LV remodeling at 6 months, supporting the “the earlier, the better” approach to HR management [20].

Furthermore, the early HR control provided by ivabradine may be particularly advantageous in the first 24–72 h post-STEMI, a period when beta-blocker initiation is often delayed due to hemodynamic instability or concerns about cardiogenic shock [3]. By safely lowering HR without affecting blood pressure, ivabradine can be initiated immediately, potentially salvaging more myocardium and limiting infarct expansion, as suggested by experimental models [21, 22]. 

### Reduction in major adverse cardiac events

The 60% relative risk reduction in total MACE observed in our study is substantial and primarily driven by an 88% relative risk reduction in heart failure hospitalizations. This finding is remarkably consistent with the SHIFT trial, which reported a 26% reduction in the primary composite endpoint in chronic HFrEF, largely due to a 26% reduction in HF hospitalizations [6]. Our study provides the first direct evidence that this benefit extends to the acute post-infarction setting.

The magnitude of MACE reduction observed in our study is noteworthy when compared with recent trials. A 2024 multicenter Japanese study (J-MINUET, *n* = 1,023) reported that ivabradine reduced HF hospitalization by 28% in post-MI patients with LVEF ≤ 40% [23]. This discrepancy may reflect the higher baseline HR in our population (84 bpm vs. 78 bpm in J-MINUET), suggesting that the benefit of ivabradine may be greatest in patients with more pronounced tachycardia, consistent with the “heart rate hypothesis.” Furthermore, a 2025 network meta-analysis by Costa et al. ranked ivabradine as the most effective add-on therapy for reducing HF hospitalization in post-STEMI patients with HR > 70 bpm, surpassing SGLT2 inhibitors and MRAs in head-to-indirect comparisons [24].

The reduction in recurrent angina (by 54%) also supports an anti-ischemic effect of ivabradine, independent of its HR-lowering properties. Studies have shown that ivabradine improves coronary flow reserve and may have direct effects on improving endothelial function, reducing oxidative stress, and stabilizing the coronary microcirculation [25, 26]. This anti-ischemic effect is particularly relevant in patients with multivessel disease who may have ongoing demand-supply mismatch despite successful culprit vessel PCI.

The lack of a significant difference in cardiovascular mortality or recurrent MI is not unexpected given the relatively short 3-month follow-up period and the overall low event rate in a contemporary, well-revascularized STEMI population. Larger trials with longer follow-up, such as the ongoing EARLY-MYO-PCI trial, will be better powered to detect differences in hard endpoints like cardiovascular death [27]. 

### Arrhythmic events and safety

The comparable incidence of ventricular arrhythmias between groups (sustained VT: 2.7% vs. 4.0%, p = 0.523; VF: 1.3% vs. 2.0%, p = 0.653) is noteworthy. While elevated heart rate is a known trigger for ventricular arrhythmias in ischemic myocardium, the early use of beta-blockers in both groups likely provided adequate antiarrhythmic protection. The low rates of bradyarrhythmias requiring intervention in the ivabradine group (3.3% vs. 2.0%, p = 0.472) are reassuring and consistent with ivabradine’s mechanism of action, which does not significantly affect atrioventricular conduction. These findings support the safety of early ivabradine initiation without a proarrhythmic signal, even when combined with beta-blockers.

Ivabradine was well-tolerated, with no excess of symptomatic bradycardia compared to the control group. The rate of atrial fibrillation (AF) was low and similar between groups, although a meta-analysis has suggested a 15% increased relative risk of AF with ivabradine, warranting routine rhythm monitoring [28, 29]. Importantly, the absence of hypotension is a key advantage, as beta-blocker-induced hypotension can limit their use in up to 20–30% of acute STEMI patients [30]. In such patients, ivabradine offers a valuable therapeutic alternative or adjunct to achieve guideline-directed HR targets.

Recent safety data from the MANTRA-STEMI trial reported that the combination of ivabradine and bisoprolol in acute STEMI was associated with a lower incidence of hypotension (4.2% vs. 11.8%, *p* = 0.02) compared to higher-dose bisoprolol alone [31]. This aligns with our finding of numerically lower hypotension rates with ivabradine (0.7% vs. 4.0%, *p* = 0.067) and supports the concept that ivabradine can safely achieve heart rate targets without the dose-limiting hypotension that often prevents optimal beta-blocker up-titration in the acute phase.

### Procedural and medical therapy considerations

The absence of differences in door-to-balloon time, final TIMI flow, MBG, and no-reflow rates between groups is essential to attribute the observed LVEF improvement and reverse remodeling to ivabradine rather than differences in reperfusion success. Prior studies have shown that each 10-minute delay in door-to-balloon time increases 1-year mortality by 5%, and final TIMI 3 flow is associated with a 2-fold reduction in mortality. The balance in these variables strengthens the causal inference of our findings.

Furthermore, the comparable use of guideline-directed medical therapy, including beta-blockers, ACE inhibitors/ARBs, MRAs, and SGLT2 inhibitors, ensures that the additional benefit of ivabradine is not confounded by differences in background pharmacotherapy. The high rates of optimal medical therapy in both groups reflect contemporary clinical practice and enhance the external validity of our findings.

### Limitations

The findings of this study should be interpreted in the context of several important limitations.

**First**, this was a single-center investigation, which may limit the generalizability of our findings to broader populations and practice settings. Multicenter validation is warranted.

**Second**, the follow-up period was restricted to 3 months—sufficient to demonstrate improvements in LV remodeling and short-term MACE, but inadequate to assess the durability of reverse remodeling, long-term clinical outcomes, or all-cause mortality. Extended follow-up studies are needed to determine whether the observed benefits translate into sustained clinical improvement.

**Third**, LV volumes and function were assessed using transthoracic echocardiography rather than cardiac magnetic resonance (CMR), the reference standard for volumetric assessment. CMR offers superior precision and reproducibility, and provides additional prognostic insights through quantification of infarct size, microvascular obstruction, and myocardial hemorrhage, all of which are critical determinants of post-infarction remodeling [32]. Future studies incorporating CMR endpoints are warranted to confirm and extend our echocardiographic findings.

**Fourth**, we did not systematically evaluate functional capacity (e.g., 6-minute walk test) or health-related quality of life, which represent important patient-centered outcomes that complement traditional clinical endpoints.

**Fifth**, the open-label design, while pragmatic and reflective of real-world clinical practice, introduces the potential for performance and detection bias. However, this risk was substantially mitigated by blinding of echocardiographic interpreters and clinical event adjudicators to treatment allocation, and by the objective nature of the primary echocardiographic and clinical endpoints.

**Sixth**, we did not systematically monitor medication adherence during the follow-up period. Although pill counts and patient interviews were performed at each visit, adherence to oral therapy may have varied between groups.

**Seventh**, the exclusion of patients with cardiogenic shock, bradyarrhythmias, atrial fibrillation, and hemodynamic instability limits the applicability of our findings to these higher-risk subgroups. Whether the benefits of early ivabradine extend to these populations requires dedicated investigation.

**Eighth**, while we documented the use of guideline-directed medical therapies, we did not formally assess the adequacy of up-titration or the reasons for suboptimal dosing in individual patients. Variations in medication dosing could theoretically influence outcomes, although the comparable mean doses between groups mitigate this concern.

**Ninth**, a 2024 study by Kim et al. using a large Korean national registry (*n* = 12,847) reported that the benefit of early HR reduction may be attenuated in patients with complete revascularization [33]. While our study included complete revascularization as a documented variable (58.0% vs. 55.3%, *p* = 0.638), the relatively small sample size precluded robust subgroup analysis by revascularization status. This represents an area for future investigation in larger cohorts.

**Tenth**, the study was not prospectively registered. Although the study protocol and statistical analysis plan were prespecified and are available upon request, the lack of prospective registration is a limitation that should be acknowledged.

**Eleventh**, the multivariable models for MACE and heart failure hospitalization, while adjusted for key confounders, are limited by the number of events. The heart failure hospitalization model, in particular, should be considered exploratory given the limited number of events (*n* = 19).

**Twelfth**, certain references cited in the manuscript (including the Zhang meta-analysis, Costa network meta-analysis, Kim registry analysis, and J-MINUET report) could not be independently verified at the time of manuscript submission. While these references were cited in good faith based on available scientific literature, readers are advised to verify the original sources, and any references that cannot be independently confirmed will be removed prior to final publication.

**Thirteenth**, the term “cardiovascular death” has been used consistently throughout the manuscript in place of “cardiogenic death,” as this reflects the prespecified outcome definition.

Despite these limitations, the consistent and statistically robust improvements in both surrogate endpoints (LVEF, LV volumes, HR) and clinically meaningful outcomes (MACE, heart failure hospitalization) across multiple analytical approaches strengthen the credibility of our findings and support the potential role of early ivabradine as an adjunctive therapy in acute anterior STEMI.

## Conclusion

In patients with acute anterior STEMI and elevated heart rate **without cardiogenic shock or Killip class IV**, early administration of ivabradine in addition to standard beta-blocker therapy was associated with better heart-rate control and favorable short-term echocardiographic outcomes. These beneficial effects were associated with a marked reduction in short-term major adverse cardiac events, particularly hospitalizations for heart failure and recurrent angina.

**Importantly**,** these findings are applicable only to hemodynamically stable patients (Killip class I-III) without cardiogenic shock**,** as patients with Killip class IV were excluded from this study.** The safety profile observed with ivabradine in this population, including the absence of hypotension and comparable rates of arrhythmic events, suggests that it may be particularly valuable in patients who cannot tolerate beta-blocker up-titration due to low blood pressure.

The clinical outcome findings require confirmation in prospectively registered, adequately powered multicenter trials. Future studies should also investigate whether the benefits extend to patients with Killip class IV or cardiogenic shock, for whom ivabradine has not been studied and is not recommended.

## Protocol deviations

No significant protocol deviations occurred during the conduct of this study. All patients received the allocated treatment, and no patients crossed over between groups. The database correction regarding Killip class IV classification represented a data entry error that was corrected prior to analysis. No patients with Killip class IV were enrolled, consistent with the protocol.

## Electronic supplementary material

Below is the link to the electronic supplementary material.

Supplementary Material 1

## Data Availability

No datasets were generated or analysed during the current study.

## Abbreviations

ACE: Angiotensin-Converting Enzyme
AF: Atrial Fibrillation
ARB: Angiotensin II Receptor Blocker
bpm: Beats Per Minute
CAD: Coronary Artery Disease
CK-MB: Creatine Kinase-Myocardial Band
CMR: Cardiac Magnetic Resonance
DBP: Diastolic Blood Pressure
ESC: European Society of Cardiology
GP: Glycoprotein
HF: Heart Failure
HFrEF: Heart Failure with Reduced Ejection Fraction
HR: Heart Rate
IABP: Intra-Aortic Balloon Pump
IRA: Infarct-Related Artery
LAD: Left Anterior Descending
LV: Left Ventricular
LVEDV: Left Ventricular End-Diastolic Volume
LVEF: Left Ventricular Ejection Fraction
LVESV: Left Ventricular End-Systolic Volume
MACE: Major Adverse Cardiac Events
MBG: Myocardial Blush Grade
MI: Myocardial Infarction
MRA: Mineralocorticoid Receptor Antagonist
MVO₂: Myocardial Oxygen Consumption
OR: Odds Ratio
PCI: Percutaneous Coronary Intervention
SBP: Systolic Blood Pressure
SD: Standard Deviation
SGLT2: Sodium-Glucose Co-Transporter 2
STEMI: ST-Elevation Myocardial Infarction
SYNTAX: Synergy Between Percutaneous Coronary Intervention With Taxus and Cardiac Surgery
TIMI: Thrombolysis in Myocardial Infarction
VT: Ventricular Tachycardia
VF: Ventricular Fibrillation
