# Supplementary material for: Early administration of ivabradine in patients with acute anterior ST-elevation myocardial infarction: a prospective, randomized, open-label study on in-hospital and short-term major adverse cardiac events and left ventricular function
Source: Egypt Heart J. 2026 Sep 22;78:72. doi: 10.1186/s43044-026-00782-z (PMC13597995; doi:10.1186/s43044-026-00782-z)
Supplement: Supplementary file 1 [file 43044_2026_782_MOESM1_ESM.docx]

**Supplementary Material**

**Supplementary Table S1: Complete-Case Sensitivity Analysis for Primary Outcomes**

| Outcome | Ivabradine Group (n = 142) | Control Group (n = 143) | Adjusted Difference (95% CI) | p-value |
| --- | --- | --- | --- | --- |
| LVEF (%), mean ± SD | 51.96 ± 6.09 | 49.79 ± 4.47 | + 2.17 (1.24 to 3.10) | < 0.001 |
| LVEDV (mL), mean ± SD | 83.65 ± 8.76 | 120.67 ± 10.15 | -37.02 (-39.45 to -34.59) | < 0.001 |
| LVESV (mL), mean ± SD | 41.06 ± 5.80 | 47.14 ± 5.42 | -6.08 (-7.42 to -4.74) | < 0.001 |
| HR (bpm), mean ± SD | 62.31 ± 1.79 | 76.13 ± 3.54 | -13.82 (-14.48 to -13.16) | < 0.001 |

**Supplementary Table S2: Propensity Score Matching Analysis (Exploratory)**

**Purpose:** This post-hoc propensity score matching analysis was performed as an exploratory sensitivity analysis to confirm the robustness of our findings. The primary analysis was the intention-to-treat analysis presented in the main manuscript.

**Methodology:**

- Propensity scores calculated using logistic regression including: age, sex, hypertension, diabetes, hyperlipidemia, baseline HR, baseline LVEF, Killip class, door-to-balloon time, multivessel disease, and baseline TIMI flow
- 1:1 nearest neighbor matching with caliper of 0.2
- 135 matched pairs identified (15 patients from each group excluded due to lack of suitable match)

| Variable | Ivabradine Group (n = 135) | Control Group (n = 135) | p-value | Standardized Difference |
| --- | --- | --- | --- | --- |
| **Age (years), mean ± SD** | 61.85 ± 4.31 | 61.72 ± 4.18 | 0.798 | 0.031 |
| **Male sex, n (%)** | 74 (54.8%) | 72 (53.3%) | 0.804 | 0.030 |
| **Hypertension, n (%)** | 68 (50.4%) | 70 (51.9%) | 0.808 | -0.030 |
| **Diabetes Mellitus, n (%)** | 49 (36.3%) | 47 (34.8%) | 0.798 | 0.031 |
| **Hyperlipidemia, n (%)** | 25 (18.5%) | 27 (20.0%) | 0.760 | -0.038 |
| **Current smoker, n (%)** | 58 (43.0%) | 56 (41.5%) | 0.804 | 0.030 |
| **Baseline HR (bpm), mean ± SD** | 83.59 ± 4.88 | 83.92 ± 4.75 | 0.572 | -0.068 |
| **Baseline SBP (mmHg), mean ± SD** | 128.4 ± 15.2 | 127.9 ± 14.8 | 0.781 | 0.033 |
| **Baseline DBP (mmHg), mean ± SD** | 78.6 ± 10.1 | 78.2 ± 9.8 | 0.738 | 0.040 |
| **Baseline LVEF (%), mean ± SD** | 47.98 ± 5.12 | 47.92 ± 5.08 | 0.924 | 0.012 |
| **Baseline LVEDV (mL), mean ± SD** | 101.38 ± 13.76 | 100.82 ± 12.68 | 0.725 | 0.043 |
| **Baseline LVESV (mL), mean ± SD** | 51.88 ± 7.92 | 52.14 ± 8.86 | 0.799 | -0.031 |
| **Killip Class ≥ II, n (%)** | 22 (16.3%) | 24 (17.8%) | 0.743 | -0.040 |
| **Door-to-balloon time (min), mean ± SD** | 58.2 ± 12.4 | 58.6 ± 12.9 | 0.793 | -0.032 |
| **Proximal LAD lesion, n (%)** | 88 (65.2%) | 86 (63.7%) | 0.801 | 0.031 |
| **Final TIMI flow 3, n (%)** | 124 (91.9%) | 122 (90.4%) | 0.669 | 0.052 |
| **MBG 2–3, n (%)** | 119 (88.1%) | 117 (86.7%) | 0.723 | 0.043 |
| **Multivessel disease, n (%)** | 48 (35.6%) | 50 (37.0%) | 0.804 | -0.030 |
| **Complete revascularization, n (%)** | 78 (57.8%) | 76 (56.3%) | 0.803 | 0.030 |
| **SYNTAX score, mean ± SD** | 21.5 ± 8.5 | 21.8 ± 8.9 | 0.773 | -0.035 |
| **Beta-blocker use, n (%)** | 131 (97.0%) | 132 (97.8%) | 0.693 | -0.049 |
| **ACE/ARB use, n (%)** | 124 (91.9%) | 123 (91.1%) | 0.823 | 0.027 |
| **MRA use, n (%)** | 38 (28.1%) | 36 (26.7%) | 0.790 | 0.033 |
| **SGLT2 inhibitor use, n (%)** | 56 (41.5%) | 54 (40.0%) | 0.805 | 0.030 |

*Abbreviations: HR, heart rate; SBP, systolic blood pressure; DBP, diastolic blood pressure; LVEF, left ventricular ejection fraction; LVEDV, left ventricular end-diastolic volume; LVESV, left ventricular end-systolic volume; LAD, left anterior descending artery; TIMI, Thrombolysis in Myocardial Infarction; MBG, myocardial blush grade; ACE, angiotensin-converting enzyme; ARB, angiotensin receptor blocker; MRA, mineralocorticoid receptor antagonist; SGLT2, sodium-glucose co-transporter 2; SD, standard deviation.*

**Supplementary Table S3: Time-to-Event Analysis for MACE Outcomes (Cox Proportional Hazards)**

| Outcome | Ivabradine Group (n = 150) | Control Group (n = 150) | Adjusted HR (95% CI) | p-value |
| --- | --- | --- | --- | --- |
| Total MACE | 19 (12.7%) | 48 (32.0%) | 0.35 (0.21–0.58) | < 0.001 |
| Heart Failure Hospitalization | 2 (1.3%) | 17 (11.3%) | 0.12 (0.03–0.48) | 0.003 |
| Recurrent MI | 4 (2.7%) | 5 (3.3%) | 0.80 (0.22–2.93) | 0.735 |
| Ischemia-driven Revascularization | 6 (4.0%) | 8 (5.3%) | 0.75 (0.27–2.11) | 0.581 |
